# The reverse genetics of HTLV-1 infected patients

**DOI:** 10.1186/1742-4690-8-S1-A193

**Published:** 2011-06-06

**Authors:** Luiz C Alcantara, Izabela Bialuk, Maria F de Castro-Amarante, Cody Buchmann, Sebastien Chevalier, Risaku Fukumoto, Vibeke Andresen, Cynthia Pise-Masison, Steven Jacobson, Bernardo Galvao-Castro, Antoine Gessain, Genoveffa Franchini

**Affiliations:** 1 Animal Models and Retroviral Vaccines Section, National Cancer Institute, Bethesda, MD, 20892, USA; 2 Oswaldo Cruz Foundation Salvador, Bahia, Brazil; 3 HTLV Center/ Bahia School of Medicine and Public Health, Salvador, Bahia, Brazil; 4 Department of General and Experimental Pathology, Medical University of Białystok, Białystok, 15-222, Poland; 5 Unité de Virologie Humaine, Département de Biologie, Ecole Normale Supérieure Lyon,Lyon, France; 6 Viral Immunology Section, Neuroimmunology Branch, National Institute of Neurological Disorders and Stroke, Bethesda, MD, 20892, USA; 7 Unité d’Epidémiologie et Physiopathologie des Virus Oncogènes, Institut Pasteur, Paris, France

## 

The HTLV-1 ORF-1-encoded p12 protein induces T-cell activation and proliferation, while the cleaved p8 protein downregulates TCR signaling. In this study, we investigated whether there is a correlation between genetic variation within ORF-1 and the clinical status or proviral load in HTLV-1 infected individuals. The ORF-1 gene was amplified by PCR from PBMCs of 163 HTLV-1-infected patients (85 carriers, 78 HAM/TSP) from different geographical regions and a total of 1,640 clones were sequenced. The majority of the patients (73%) carried mutations in ORF-1 that resulted in the expression of more p12 than p8 (50% Carriers and 50% HAM/TSP). The highest genetic variability within ORF-1 was found in the two transmembrane domains of the protein. Of interest, a subclass of mutations was found more frequently in HAM/TSP patients compared to Carriers. While higher proviral loads were found in HAM/TSP patients compared to Carriers (p=0.0001), no correlation between proviral load and the ORF-1 isoform expressed was observed (mainly p12, both p12 and p8, or mainly p8). Currently experiments are aimed at determining the significance of these mutations in ORF-1 function in regard to T-cell activation and proliferation. In addition, mutations will be characterized for their role in viral infectivity and transmission rates ex vivo and in a rhesus macaque model. Determining the effects of ORF-1 mutants on viral replication, spread and latency will provide insight into the pathogenesis of HTLV-1 infection.

